# BIM and Computer Vision-Based Framework for Fire Emergency Evacuation Considering Local Safety Performance

**DOI:** 10.3390/s21113851

**Published:** 2021-06-02

**Authors:** Hui Deng, Zhibin Ou, Genjie Zhang, Yichuan Deng, Mao Tian

**Affiliations:** 1 School of Civil Engineering and Transportation, South China University of Technology, Guangzhou 510641, China; hdeng@scut.edu.cn (H.D.); 201921007707@mail.scut.edu.cn (Z.O.); 201721007556@mail.scut.edu.cn (G.Z.); 2 Key Laboratory of Urban Land Resources Monitoring and Simulation, Ministry of Natural Resources, Shenzhen 518034, China; 3 State Key Laboratory of Subtropical Building Science, Guangzhou 510641, China; 4 Sonny Astani Department of Civil and Environmental Engineering, Viterbi School of Engineering, University of Southern California, Los Angeles, CA 90007, USA; tianmao@usc.edu

**Keywords:** computer vision, BIM, fire hazard, emergency evacuation, indoor positioning, local safety performance

## Abstract

Fire hazard in public buildings may result in serious casualties due to the difficulty of evacuation caused by intricate interior space and unpredictable development of fire situations. It is essential to provide safe and reliable indoor navigation for people trapped in the fire. Distinguished from the global shortest rescue route planning, a framework focusing on the local safety performance is proposed for emergency evacuation navigation. Sufficiently utilizing the information from Building Information Modeling (BIM), this framework automatically constructs geometry network model (GNM) through Industry Foundation Classes (IFC) and integrates computer vision for indoor positioning. Considering the available local egress time (ALET), a back propagation (BP) neural network is applied for adjusting the rescue route according to the fire situation, improving the local safety performance of evacuation. A campus building is taken as an example for proving the feasibility of the framework proposed. The result indicates that the rescue route generated by proposed framework is secure and reasonable. The proposed framework provides an idea for using real-time images only to implement the automatic generation of rescue route when a fire hazard occurs, which is passive, cheap, and convenient.

## 1. Introduction

According to the report released by the Ministry of Emergency Management of China, 1045 people were killed by fire in residential and public buildings in 2019, accounting for 78.3% of the total fire fatalities [1], which clearly reveals the urgent need of reducing the fire hazards in public buildings [2,3]. Due to the suddenness of fire and the intricate interior space of public buildings, emergency and evacuation are full of uncertainties in a fire scenario [4,5], making it vital to provide a safe and reliable indoor rescue route for the evacuees [6,7]. To offer guidelines for indoor evacuees in case of fire emergency, this paper presents a framework for generation of reliable rescue route, considering the safety performance of evacuation in fire scenario.

In the field of indoor navigation, the prior information provided by Building Information Modeling (BIM) has been gradually applied and expended [8,9]. The present research mainly utilized BIM models for extraction of indoor road network of buildings, preparing for the path planning process [10]. Furthermore, for accurate indoor rescue routing, indoor positioning techniques are essential and have been extensively studied, including radio frequency identification, Bluetooth, wireless local area network (WLAN), and visible light [4,7,11]. However, these active indoor positioning techniques, which are relatively less reliable in a fire scenario, are relatively complex and expensive. The ability of BIM for providing rich prior information has been underutilized [12,13,14]. Furthermore, as for conducting indoor navigation, most of the existing research studies adopted global shortest path planning method, which is widely applied in normal indoor navigation, ignoring the safety performance of the generated rescue route [15]. Furthermore, there have been many significant studies on the indoor navigation of robots, such as vision-and-language navigation and remote embodied visual [16,17,18,19], which have provided ideas for indoor navigation and rescue routing of human beings. It is worth noting that the safety performance of the rescue route is sometimes more significant than its simple distance under fire scenario [5,9].

In this paper, a framework for indoor rescue routing has been constructed, emphasizing three essential steps of evacuation navigation. Firstly, exploring the information integration capability of BIM model, the method for extracting indoor road network is improved to fully consider the topological and semantic information from BIM. Secondly, BIM model is integrated with computer vision technology to realize a passive and relatively cheap indoor positioning function for evacuation navigation. Since it is difficult to take the spread of the fire into account at short notice, the generation of a rescue route is improved by a machine learning-based approach with consideration of safety performance. In brief, the research proposes a framework for indoor fire emergency navigation where the reliability and safety performance of rescue route generation are emphasized.

The proposed framework for fire emergency evacuation navigation includes indoor geometry network model (GNM) generation module, indoor positioning module and indoor rescue route planning module. Employing the rich information of BIM, this framework automatically constructs GNM through Industry Foundation Classes (IFC) and integrates computer vision for indoor positioning [10]. BIM restores rich topological and semantic information between construction components and support full life-cycle data sharing [20]. The indoor GNM generation module utilizes BIM as the model input and IFC as the intermediate format to extract indoor topological and semantic information, generating a reliable GNM for indoor evacuation navigation [21,22]. In addition, the proposed BIM and computer vision-based indoor positioning module provides accurate local positioning of the interior, determining the spatial relationship between evacuees’ position and GNM. Furthermore, it is a reliable passive indoor positioning method which avoids the risk that the active indoor positioning technology may fail due to fire. Once obtaining the positioning information, the rescue route is generated in a timely manner for prompt evacuation navigation. Since the development of fire is unpredictable [15], the planning of rescue route should not only consider the length of rescue route, but also take account of the location and development of the fire [23,24]. By combining spatial relationship of GNM and precise indoor positioning information, a back propagation (BP) neural network is applied to adjust the rescue route according to the fire situation. Considering global egress time tends to lead to the safety and reliability of rescue route being ignored [25]; in the process of fire emergency evacuation navigation, focusing on available local egress time (ALET) helps to improve the local safety performance of a rescue route compared to available global egress time, which does not consider the development of the fire situation, providing more reliable routing for evacuation.

This research presents a general framework considering the safety performance of evacuation for the automatic generation of an indoor rescue route, covering a series of processes from the input of BIM and images to the output of rescue route. It only requires real-time images to implement the automatic generation of the rescue route in a fire scenario, which is cheap and convenient. The rich prior information contained in BIM has been applied and an indoor passive positioning method is proposed to provide location information for navigation. Furthermore, the framework applies a BP neural network based on ALET for rescue route planning. Compared with traditional algorithm for path planning [11,21,24], the proposed algorithm not only improves the safety performance of a rescue route, but also shortens the required time for operating. A campus building is taken as an example for proving the feasibility of the framework proposed. The result indicates that the rescue route generated by the proposed framework is more secure and reasonable.

## 2. Related Work

Researchers have made more attempts in employing BIM/IFC for emergency evacuation. At present, the BIM model is mainly applied for simulation and the results are used to further optimize the building design. Since the role of BIM lies in information sharing, some research studies have tried to utilize the rich geometric and semantic information in BIM and combine BIM with indoor positioning technologies to generate a rescue route. However, currently indoor positioning methods mostly rely on pre-arranged sensors, which have a high deployment cost and fail easily in fire scenarios. Therefore, a more accurate and convenient indoor positioning method is needed. It is also worth noting that the shortest path in a fire scenario may not be the optimal one. The safety and reliability of the rescue route should be fully considered to establish a reasonable evacuation navigation strategy.

### 2.1. Fire Emergency Management Based on BIM

The application of BIM technology in emergency management mainly focuses on evacuation simulation and fire dynamic simulation. Ma and Wu (2020) [26] discussed the theoretical framework of BIM technology applied in emergency evacuation simulation, and provided reference suggestions for BIM model’s design depth and parameterized standards of emergency management. Jeon (2015) [27] utilized the existing BIM model and combined commercial software to simulate and analyze the fire development and personnel evacuation under different working conditions. Based on the comparison of the simulation time of the two situations, the optimized architectural design plan was proposed. Bi et al. (2013) [28] analyzed the defects of the existing fire emergency plans based on the survey results, and proposed a dynamic emergency method based on BIM. The visual path planning program with the evacuation path simulation software has promoted the application of BIM in fire emergency planning.

However, research studies based on simulation software provide only an optimized solution for building fire performance in the design or construction phase, without proper guidance for personnel evacuation in a fire scenario. The visual characteristics of the BIM model provides accurate information on the interior of the building, so that they can obtain a safe and reliable rescue route while fully perceiving the indoor environment, improving the efficiency of rescue. Therefore, some scholars have gradually been focusing on the spatial information of BIM, combined with sensor-based positioning technology to establish the emergency navigation system for evacuees [8]. However, there is still a lack of reliable research on passive indoor location technology, and few studies have emphasized the safety performance of rescue route when navigating the evacuation path for evacuees.

### 2.2. GNM Extraction

**Figure 1:**
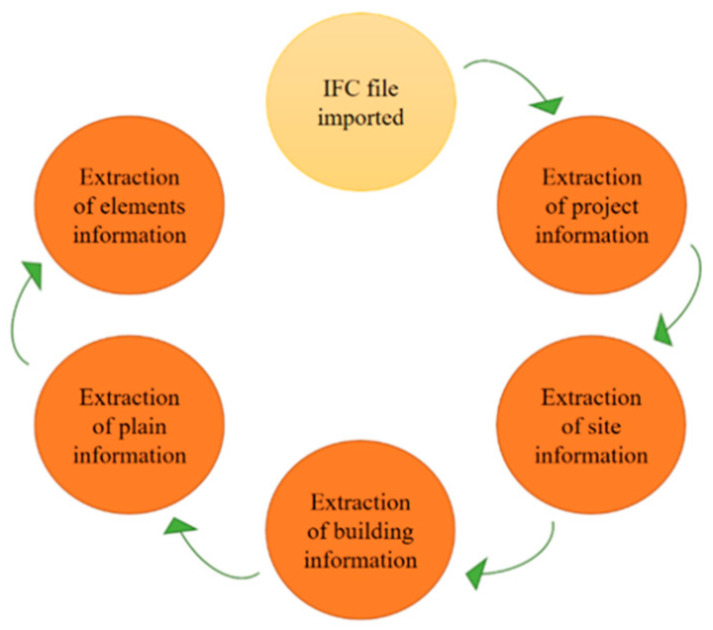
Extraction process of IFC.

IFC has provided a solution for the standardization of BIM information storage applications in engineering construction, as shown in Figure 1. Describing the definition of various entities and regulating industry standards, it can effectively solve the problem of heterogeneous data fusion and global data sharing in distributed engineering by establishing an IFC standard system [29].

For extracting navigation networks through BIM, Lin and Lin (2018) [30] presented an approach called i-GIT to produce a graph-based indoor network including floor-level and non-level paths from IFC-based building models, requiring only the geometric information of IFC data models to automatically identify indoor space boundaries. Kim and Lee (2019) [8] established a framework to automatically analyze, generate, and visualize the evacuation paths of multiple crews in 4D BIM, considering construction activities and site conditions at the specific project schedule. Hamieh et al. (2020) [21] proposed a system, called BiMov, dedicated to automatically determining plan paths in complex buildings based on a digital mock-up. Fu et al. (2020) [10] generated straight skeleton-based navigation networks with IFC for indoor wayfinding. Teo and Cho (2016) [29] extracted building information from IFC files and established corresponding spatial topological relations to generate navigation networks. Peng et al. (2019) [9] constructed the indoor GNM by extracting the building information from BIM, and applied the artificial neural network algorithm for path planning in fire scenarios.

### 2.3. Indoor Positioning and Computer Vision

As the foundation of indoor path planning in a fire scenario, indoor positioning refers to the process of understanding the position and orientation of people or objects in an indoor environment, determining the spatial relationship between evacuees and GNM. Since it is formidable to receive GNSS signals in an indoor environment [31], various technologies are applied to achieve indoor positioning, including radio frequency identification (RFID), Bluetooth, wireless LAN, and visible light, which require the deployment of related infrastructure [11,12,13,14]. In terms of indoor positioning integrated with BIM/IFC, Ma et al. (2018) [32] applied the Wi-Fi and geomagnetic fingerprint matching positioning method and integrated it into the BIM construction quality management system to solve the problems of missed inspection and secondary data recording during construction acceptance. Carrasco et al. (2018) [33] established a system that runs in an Android app and finds the nearest machine to a user. The system collects the received signal strength indicators (RSSIs) from low-cost Bluetooth beacons installed in the machines. The sensor inertial measurement unit (IMU) was used to locate the indoor personnel inside the building. Combined with the characteristics of the building structure in the case of a disaster, the IMU provides quick decision support ability for disaster scenarios. Li et al. (2014) [4] developed a collaborative operation platform to integrate sensor positioning information to provide reliable geometric spatial information for the sensor field, and at the same time quickly locate the evacuees through the sequence-based localization mode. The BIM spatial positioning function was used to improve the room-level positioning accuracy. La et al. (2016) [34] proposed an indoor emergency navigation method, which can accurately locate indoor personnel.

With the rapid development of computer vision in construction quality management [35,36], crack detection [37,38,39] and safety management [40,41,42], some researchers proposed to apply computer vision technology integrated with BIM for indoor navigation to overcome the high cost and signal stability requirements of above-mentioned indoor positioning technologies. Yang et al. (2010) [43] proposed a solution that uses cameras to locate and track multiple construction site workers through template matching, which achieves the identification and statistics of multiple workers from the same perspective, and real-time positioning. Gargi and Sandeep (2015) [44] proposed a computer vision-based vehicle monitoring method at the construction site, automatically performing statistics and monitoring of vehicles on the construction site. Fang et al. (2020) [45] enhanced the monocular vision technique for the localization of construction-related entities. Acharya et al. (2019) [46] proposed a visual localization approach to eliminate the requirement of image-based reconstruction of the indoor environment by geometric feature matching. A BIM and computer vision-based method for indoor positioning was presented, obtaining accurate position information with prior information in BIM.

**Figure 2:**
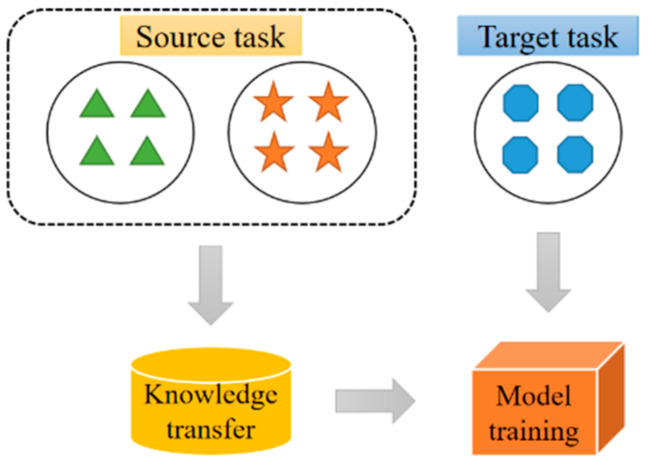
Transfer learning.

When utilizing computer vision technology for indoor positioning, transfer learning methods can be applied for effective recognition of neural network because of its better generalization and robustness in the case of small samples [47,48]. It refers to the process of improving the learning effect by transferring the knowledge learned from a specific task to a new one, as shown in Figure 2.

Scale invariant feature transform (SIFT) and perspective-n-points (PnP) problem can be utilized for realizing the pose estimation of camera, so as to achieve indoor positioning of evacuees [49,50]. SIFT can be adopted to extract and match image feature points insensitive to changes in illumination, scale, and viewing angle from the two images with its strong robustness. Furthermore, PnP problem assumes that if the internal parameters of the camera are known, through setting three or more pairs of control points, the pose of camera can be calculated according to the correspondence between its 3D space coordinates and 2D pixel coordinates with a homography transformation relationship. Let $H=H_{2}H_{1}^{-1}$, then *H* describes the correspondence between pixels of the same object, namely the homography matrix which describes the correspondence between points of two images. Only four matching feature points are needed to obtain the homography matrix.

### 2.4. Rescue Route Planning

For the research studies of fire evacuation strategy, Zhang et al. (2013) [51] proposed a probabilistic occupant evacuation model for fire emergencies using Monte Carlo methods and then integrates the model into the fire risk analysis model. Kong et al. (2017) [52] conducted an investigation of a method linking safety factors and probability of failure in building fire safety design. As can be seen, some of the research studies on indoor emergency evacuation focus on the optimization of building design results through simulation method. However, Kurniawan et al. (2018) [53] indicated that performance-based method considering the parameters of the available safe egress time (ASET) and required safe egress time (RSET) is difficult to achieve because of the lack of resources and experts in applying related software. Furthermore, the simulation software is difficult to be applied for fire evacuation guidance due to its low operational efficiency.

As for real-time indoor evacuation navigation in fire scenario, Teo and Cho (2016) [29] explored the strategy of indoor and outdoor network connections for evacuation navigation, generating the rescue route as a whole through integrating BIM and GIS. Kim and Lee (2019) [8] proposed a framework to automatically analyze, generate, and visualize the overall evacuation paths of buildings by extracting information from BIM models. In similar research studies, BIM is mostly utilized as the information source to generate GNM, and the traditional shortest path planning algorithm is adopted to select the optimal path. Mirahadi et al. (2019) [25] developed a decision-support system with ALET to offer evacuation strategies fully considering the safety and reliability of rescue route.

Previous studies tend to ignore the development trend of fire and safety of rescue routes, which may lead to incorrect evacuation navigation, waste of precious evacuation time, and even unnecessary casualties. In rescue route planning module of the proposed framework, the safety performance of rescue route is properly considered and evaluated. For considering the fire development, all of the potential ignition points and starting points of evacuation are randomly generated, based on which all of the evacuation paths are calculated. Once the optimal path is determined by the evaluation function proposed, they will be input as the samples for training the BP neural network, which has the ability to generate relatively safe rescue route. Through applying BP neural network, the shortened computing time can provide more evacuation time for the evacuees.

To summarize, the framework proposed in this article mainly covers three aspects of technological innovation, as shown in Table 1. In the phase of GNM generation, the proposed method extracts the nodes and edges from IFC file through considering its topological and semantic information, achieving a reasonable extraction process. As for indoor positioning, a passive and cheap way is accessible in the framework, because there is no requirement for installing related equipment in advance. Based on the extracted GNM and positioning result, the proposed framework generates a relatively safe rescue route for evacuees and utilizes BP neural network for shortening the computing time.

## 3. Materials and Methods

**Figure 3:**
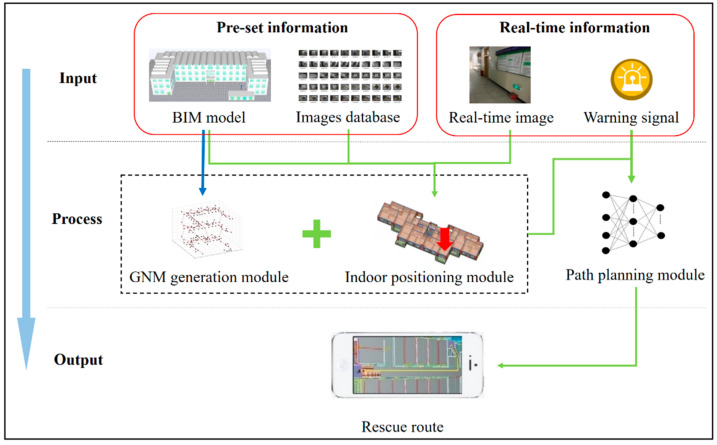
Methodology overview.

In the framework shown in Figure 3, the BIM model and images database are set as pre-set information, preparing for positioning and path planning. Real-time images and warning signal are input as real-time information for real-time positioning and path planning when a fire hazard occurs. The rich prior information contained in BIM has been applied for GNM generation and indoor positioning. Furthermore, concerning the safety performance of evacuation, this framework applies BP neural network based on ALET for rescue route planning.

### 3.1. GNM Generation Module Based on IFC

**Figure 4:**
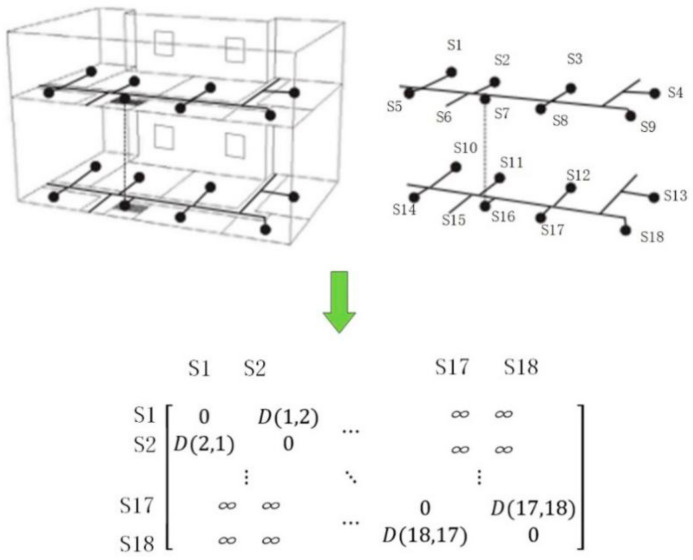
Geometric network model.

Graph theory describes the topological relationship between object through geometric principles, simplifying specific object and their relationship into nodes and edges. For the construction of indoor GNM, nodes are utilized to represent the spatial elements in the building and edges are applied to represent the topological relationship between the nodes. As shown in Figure 4, the relationship between nodes is represented by a weighted adjacency matrix D, which represents the weight of the edge. If the edge does not exist, the value in D becomes ∞, showing the impassability of the path.

The network model for indoor space information mainly includes: space nodes, horizontal paths and vertical paths. The space nodes represent the internal area of the building, including offices, classrooms and toilets. The horizontal path includes the horizontal connection of corridors, doors and corridors, and the horizontal connection of doors and room nodes. The vertical path represents the staircase connection between floors. Table 2 describes the required IFC indoor space elements.

**Figure 5:**
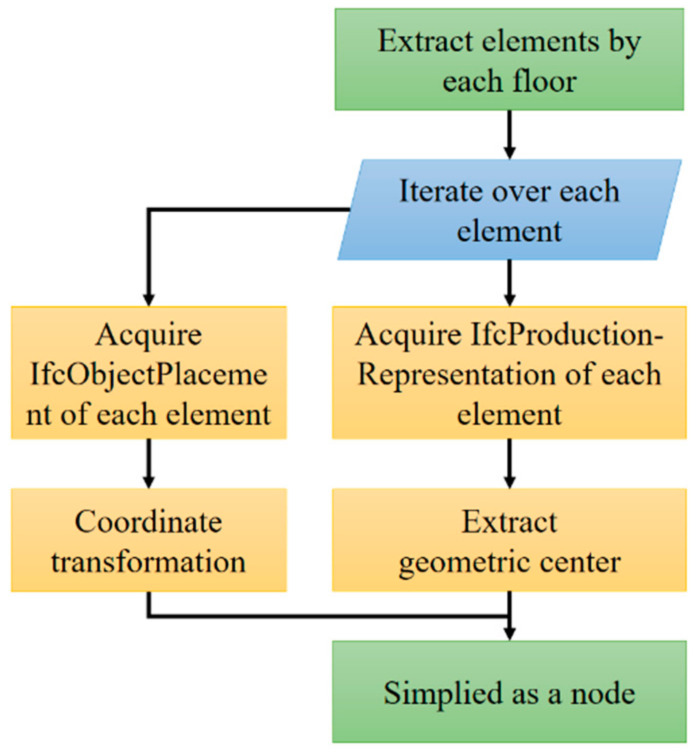
Flowchart of node generation.

For the generation of nodes, the indoor spatial elements should be extracted and simplified to obtain their spatial coordinates. The relative coordinate system is used instead of the mapping coordinate system, so that all components must be unified in the same coordinate system (IfcSite), which is realized by the conversion matrix obtained from IfcLocalPlacement and IfcAxis2Placement3D. The specific process of node generation is shown in Figure 5. The maximum and minimum values of the coordinates of the extracted vertex coordinates of the geometric model in the X and Y directions are extracted to obtain the outsourcing size of the model. The two-dimensional center point ($\left(X_{min}+X_{max}\right)/2$, $\left(Y_{min}+Y_{max}\right)/2$) is chosen as a simplified node. In order to intuitively observe the distribution of each node, the value of each node in the Z direction is reduced to the floor height.

(1) $$N=(\frac{X_{max}+X_{min}}{2},\frac{Y_{max}+Y_{min}}{2},Z_{storey})$$

**Figure 6:**
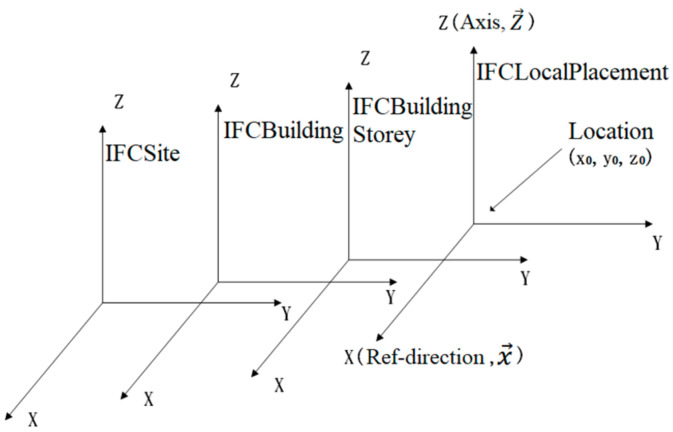
Relationship of coordinate transformation.

The coordinate positioning is realized by multi-level chain reference. For example, the local coordinate system of the floor slab refers to the floor coordinates system and the local coordinate system of the floor refers to the building coordinate system (shown in Figure 6). Therefore, there are rotation and translation relationships between different coordinate systems. The coordinate systems referenced mutually are connected through IfcAxis2Placement, where location in IfcAxis2Placement is the translation amount, and RefDirection is the unit direction vector of the *x* axis, and Axis is the unit direction vector of z axis. The direction vector of the *y* axis can be obtained from the known *x* axis and *z* axis unit direction vectors, and then the rotation matrix is obtained [54]. The specific coordinate conversion formula is as follows:

(2) $$\left[x^{\prime }\;y^{\prime }\;z^{\prime }]=\left[x\;y\;z\right]\times {\left({{\left[\vec{x}\;\vec{y}\;\vec{z}\right]}^{T}}^{-1}\right)}^{T}+[x_{0}\;y_{0}\;z_{0}\right]$$

where: $x^{\prime },y^{\prime },z^{\prime }$ represent the converted coordinates. x,y,z represent the original coordinate. $x_{0},y_{0},z_{0}$ represent the amount of translation. $\vec{x},\vec{y},\vec{z}$ represent the initial unit vectors for the *x* axis, *y* axis, and *z* axis.

The edges in the road network mainly include horizontal and vertical edges. The horizontal edges include corridor skeleton lines, the connection of stairs and corridors, the connection of corridors and doors, and the connection of rooms and doors, while the vertical edges connect the stairs nodes between floors.

- Corridor skeleton line: As the channel connecting rooms, if the geometric outline of corridor is simply reduced to a spatial node, it cannot reflect the real spatial topology information. Thus, corridor information should be extracted from the skeleton line of the corridor.
- Stair to corridor: The stair represents the vertical path connecting different floors. By extracting IfcStair, a staircase node is generated and adjacent staircase nodes are connected in sequence. The stair node is connected to the node closest to the corridor, so as to realize the connection of the vertical node and the horizontal path.
- Corridor to door: The topological relationship between corridor and door can be extracted from IfcRelSpaceBoundary. The nodes of all the doors are extracted and connected to the nearest point on the corridor route in turn.
- Room to door: The topological relationship between room and door is also recorded in the IfcRelSpaceBoundary attribute. Therefore, it is only necessary to traverse all the adjacent components in the room in IfcRelSpaceBoundary, searching for components belonging to IfcDoor and connecting the nodes.

### 3.2. Indoor Positioning Module Utilizing Computer Vision

**Figure 7:**
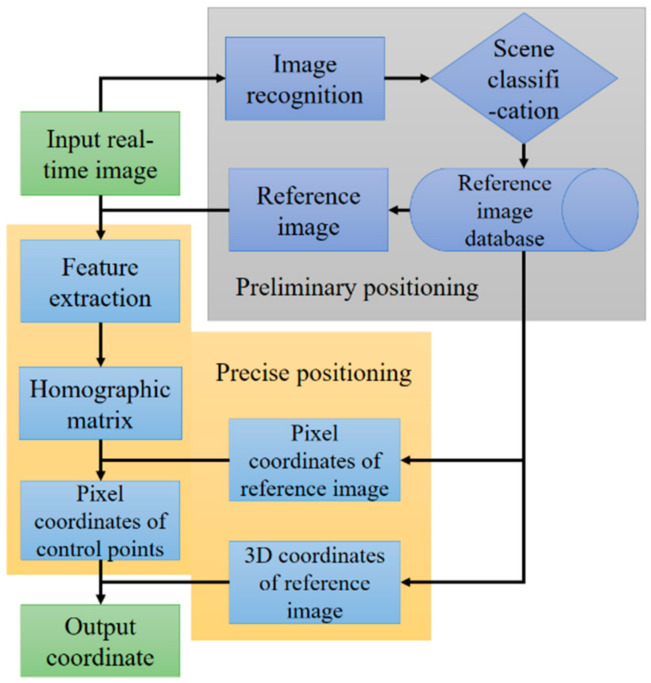
Flowchart of indoor positioning.

In this study, computer vision technology is applied to ascertain evacuees’ position in GNM. The main process of indoor positioning based on computer vision shown in Figure 7 includes: preliminary positioning through recognition of indoor images; Setting control points for specific scenes and extracting pixel coordinates and 3D coordinates of the control point from the identified specific scene; obtaining pixel coordinates after perspective transformation by SIFT algorithm; applying RPnP algorithm for precise positioning.

**Figure 8:**
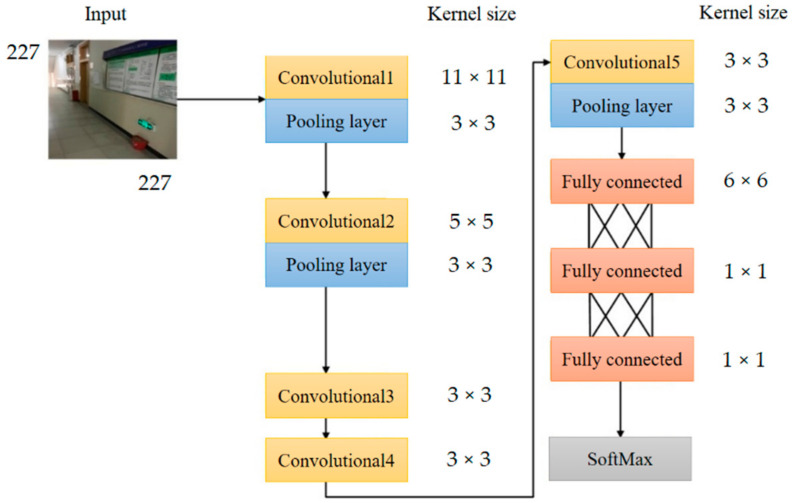
Structure of AlexNet.

Indoor scene recognition can preliminarily locate the image and determine the scene where the image belongs. In order to achieve a better recognition effect in a limited data set, the AlexNet [55] which performs well in large-scale image recognition tasks is utilized for transfer learning, with its structure shown in Figure 8. Adopting transfer learning strategy, this paper retains the convolutional layer and pooling layer of the AlexNet model trained on the large data set ImageNet, with the last three layers replaced by a fully connected layer, a softmax layer, and a classification output layer and the number of the parameters in fully connected layer equaling to the number of image categories. To extract and match image feature points insensitive to changes in illumination, scale, and viewing angle from the two images, SIFT is adopted with its strong robustness to the transformation of image scale, illumination, rotation and angle of view. The proposed framework applies RPnP algorithm [56] for obtaining the homography matrix.

According to the result of scene recognition, the reference image and control point parameters of the corresponding scene are extracted. Considering the computational efficiency, four control points for the reference images of each scene are set. Among them, the 3D coordinate system utilizes the BIM coordinate system. The corner points of the target components that are the same as the real scene are picked as the control points in the BIM model. Through the homography matrix solution and RPnP algorithm that obtain the 3D coordinates of the shooting equipment, the coordinates approximately represent the actual location of the evacuee.

### 3.3. Path Planning Applying BP Neural Network

Fire emergency evacuation is affected by many factors, such as the uncertainty of physical characteristics and psychological state of evacuees, the layout of evacuation sites, the performance of building materials, fire alarm system and emergency management ability, with a great impact on the evacuation process. The traditional algorithms for path planning [11,21,24] consider only the length of path, and take a relatively long time to run. Studies applying a traditional path-finding algorithm for rescue routing tend to ignore the safety performance of rescue route, being inconsistent with the requirement of evacuation in fire situation. Mirahadi et al. (2019) [25] proposed the ALET which measures the remaining egress time of the rest location at a specific time, reflecting the safety status of evacuees in the rest evacuation way. In rescue route planning module, the safety of the generated rescue route is guaranteed by adopting ALET as the time factor in the rescue route planning algorithm, combined with the path evaluation system. Indirectly considering the local safety performance of evacuation, the score of path *k* from node *i*, namely $L_{k}$, is calculated by Formula (3) based on the consideration of ALET, where: $l_{k}$ is the length of path *k*, $l_{max}$ and $l_{min}$ are, respectively, the longest and the shortest path length in all paths starting from node *i*.

(3) $$L_{k}=\frac{l_{k}-l_{min}}{l_{max}-l_{min}}$$

In order to balance the length and safety performance of rescue route, the path evaluation function in rescue route planning module includes two factors which, respectively, are the safety and length of the rescue route. Therefore, the evaluation formula is shown as Formula (4), where: *L* is the length fraction of the path; *E* is the safety fraction of the path; $\mu_{1}$ and $\mu_{2}$ are the corresponding weight with sum of 1.

(4) $$S=\mu_{1}L+\mu_{2}E$$

As for safety score *E*, according to the adjacency relationship of nodes in the path, it is considered to punish the node with fire point or adjacent fire point in the path. For example, the initial score of *E* is set to 1, with −0.3 for each fire point appearing in the path and −0.1 for each node adjacent next to the fire point, representing the different distance from the fire. In other words, *E* represents the safety status of the node directly, helping the path planning algorithm to avoid nodes close to the fire source and achieve safer evacuation navigation. The coefficients −0.1 and −0.3 are used to represent the impact of fire, and the specific value should be further acquired by experiments.

**Figure 9:**
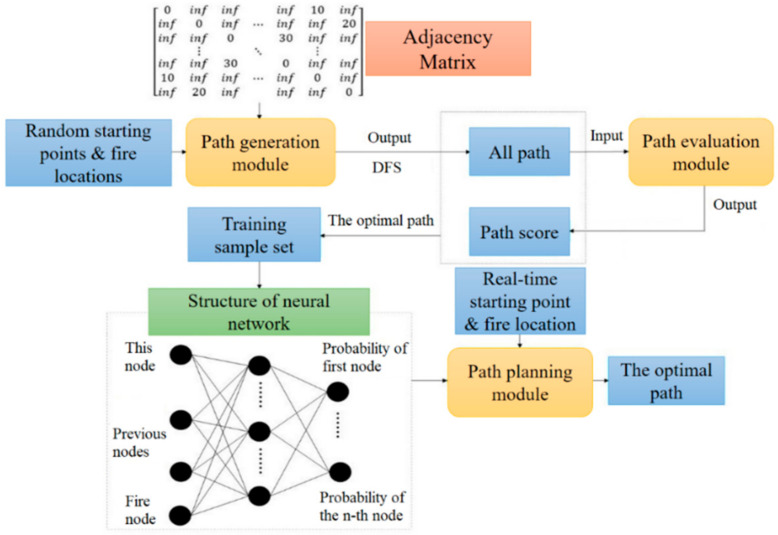
Training of rescue route planning network.

Considering the efficiency and safety of evacuation, this paper applies neural network algorithm for rescue route planning in fire evacuation scene, mainly including path generation module, path evaluation module and path planning module, shown in Figure 9. Among them, the path generation module and path evaluation module provide sample set for the training of neural network, while the path planning module plans the rescue route through the trained network. The finally resulting path planning module can be directly utilized for fire emergency navigation.

At the beginning of network training, the path generation module searches all available rescue routes for different starting points and fire points through the depth first search (DFS) algorithm. After that, all of the generated available rescue routes are evaluated by path evaluation module for scoring and determining the rescue route with best score as the training sample of the designed BP neural network. By setting a learning goal, the sample set is utilized for training. When the performance of training neural network reaches the set goal, it can be adopted for rescue route planning of fire evacuation, taking local safety performance of evacuation into account and improving the efficiency of rescue route generating.

**Figure 10:**
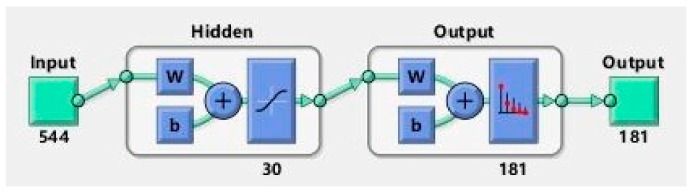
Structure of BP neural network.

A BP neural network algorithm is adopted in this paper to build a rescue route planning model. In order to generate the sample set, the starting points and fire points for the topology nodes are randomly generated by simple random sampling method with all accessible paths generated by DFS algorithm. After data training, the coordinate of starting point is input into the neural network for calculating the optimal rescue route as output. Applying BP neural network for path planning, a three-layer BP neural network structure containing a hidden layer is used as the basic structure of the prediction model, as shown in Figure 10.

After testing and comparison, in this paper, the number of neurons in the hidden layer is selected as 30, with the learning rate set as 0.5, the target training error set as 0.0001, the learning function selected as transcg, the transfer function from the input layer to the hidden layer set as Tanh, and the transfer function from the hidden layer to the output layer set as Softmax. Since the path prediction in this paper is realized through the chain prediction between nodes, every prediction can actually be regarded as a classification problem. By comparing common cost functions such as mean squared error, root mean squared error, mean absolute error and cross entropy [57,58,59], the cross entropy, which is appropriate for solving classification problems, is selected as the cost function:

(5) $$H\left(p,q\right)=-\overset{N}{\underset{i=1}{\sum }}p\left(x^{\left(i\right)}\right)logq\left(x^{\left(-i\right)}\right)$$

where, *N* represents the total number of samples, *p*(*x*) refers to the probability of the true distribution, and *q*(*x*) refers to the probability estimate calculated through the model. The performance of cost function is affected by the total number of the samples, which should be taken into account for accurate result.

## 4. Results

The proposed framework of indoor rescue route planning was tested in a three-floor campus building in South China University of Technology. Through a BIM model established according to the as-built drawings, an IFC model was generated and its class/relations extraction was implemented by IFC JAVA TOOLBOX toolkit [60]. In general, IFC models can be exported by mainstream BIM software or by some independent IFC tools. The indoor positioning and path planning process was implemented in MatlabR2018b using a personal computer with 2.30 GHz CPU and 8 GB RAM.

### 4.1. Generation of GNM

**Figure 11:**
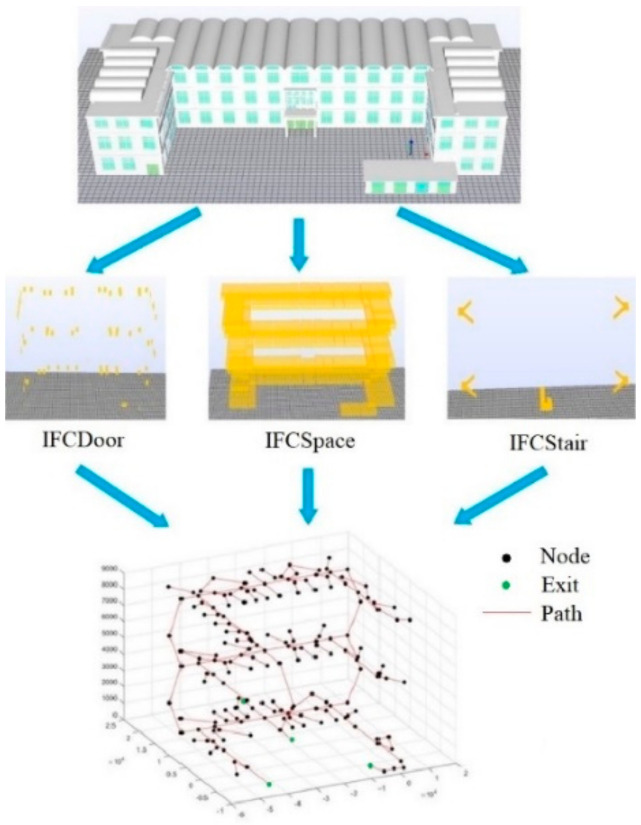
Geometric network model generation.

The prototype of IFC-to-GNM conversion for transferring information from BIM/IFC into GNM was implemented in two steps. Firstly, the JAVA TOOLBOX toolkit is applied to extract the corresponding IFC classes/relations. Secondly, in Java environment, the relationships of the elements are adopted to generate the topological primitives. The number of related elements is shown in Table 3 and the generation of GNM is shown in Figure 11.

### 4.2. Indoor Local Positioning

**Figure 12:**
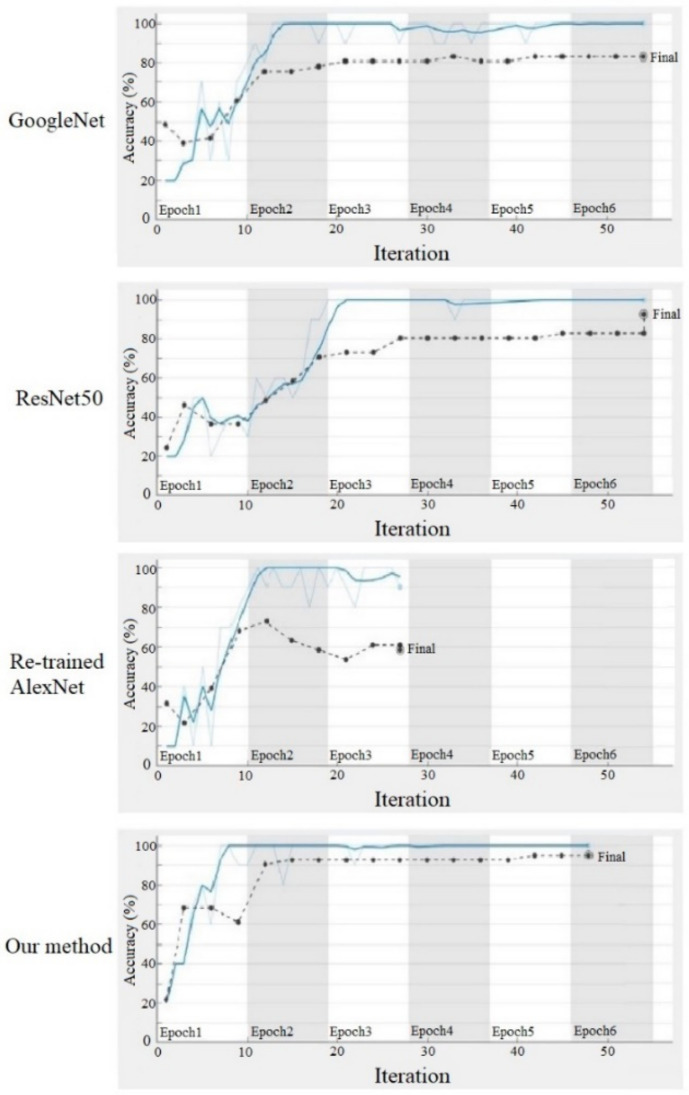
Accuracy curve of different methods.

**Figure 13:**
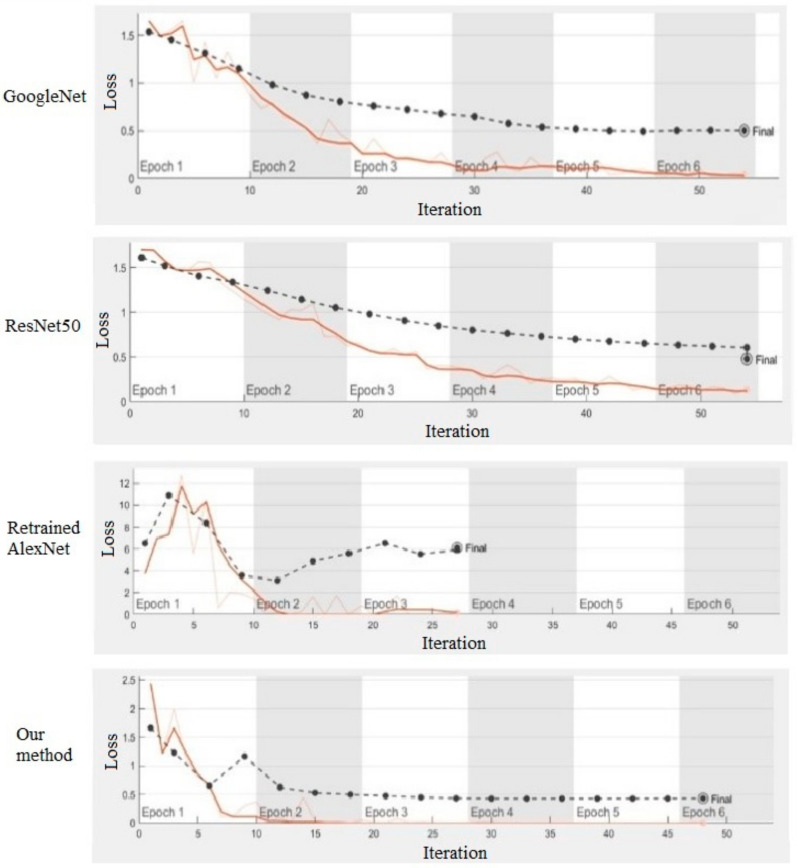
Loss curve of different methods.

The training sample set contains a total of five types of indoor scenes, each with 100 scenes. Transfer model of AlexNet is trained with a stochastic gradient descent algorithm as an optimization algorithm, and its learning rate is set to $10^{-4}$, with 70% training set and 30% test set. In order to verify the performance of transfer model, AlexNet, Googlenet and ResNet50 are applied as contracts with the accuracy and loss curve of the training process shown in Figure 12 and Figure 13.

The accuracies of different methods in test sample are, respectively, 58.54% (re-trained AlexNet), 82.93% (transfer GoogleNet), 92.68% (transfer Resnet) and 95.12% (our method). It is obvious that the accuracy of the classification method applied in this paper is significantly improved compared to other methods. In a small sample set, the retrained AlexNet network performs relatively poorly compared to the other three transfer learning models.

**Figure 14:**
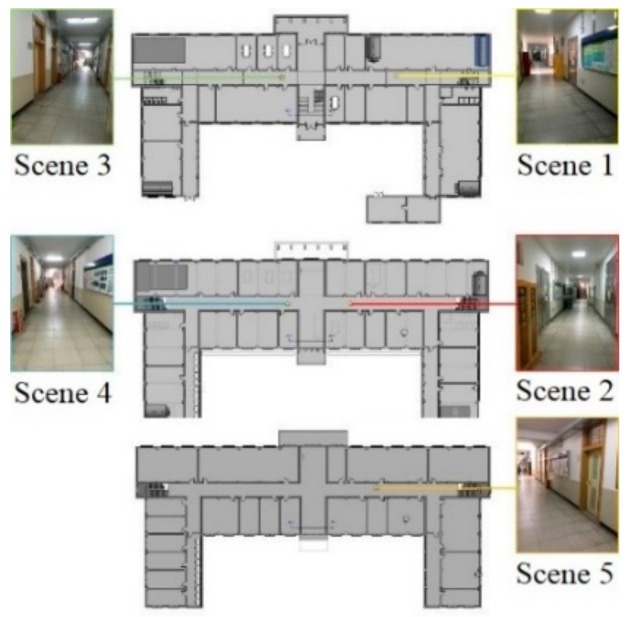
Reference images of different scenes in the building.

After the image is correctly identified, the reference image of the scene will be extracted according to the identified label and the positioning will be solved by the positioning algorithm. The true value of the positioning coordinates is measured by the Suguang OTS-632BL total station whose ranging accuracy is ±(2 + 2 × 10^−6^·D) mm, while the image is taken with an iPhone SE mobile phone whose internal parameter matrix after calibration and distortion coefficient is shown in Formulas (6) and (7). Set with four control points, the reference images of each scene are shown in Figure 14.

(6) $$\mathrm{Calibrated}\;\mathrm{Internal}\;\mathrm{Parameter}\;\mathrm{Matrix}=\left[\begin{array}{ccc} 3256.4 & 0 & 2138.8\\ 0 & 3243.6 & 1555.5\\ 0 & 0 & 1 \end{array}\right]$$

(7) $$\mathrm{Distortion}\;\mathrm{Coefficient}=\left[\begin{array}{ccc} 0.129 & -0.131 & \begin{array}{ccc} 0 & 0 & 0 \end{array} \end{array}\right]$$

**Figure 15:**
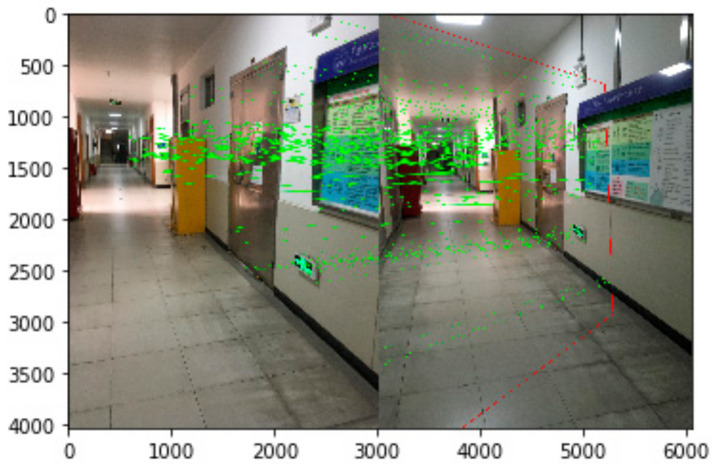
Feature matching.

Through feature extraction and matching process, as shown in Figure 15, the calculated homographic matrix is shown as Formula (8) by applying the SIFT algorithm. The coordinates of the control points after the image conversion are shown in Table 4. Combining the 3D coordinates of the control point, the positioning is solved by the RPnP algorithm to be (−7657.349, 15,607.830, 9603.302) mm, with positioning error of 1.44 m. Since value *Z* is between 8200 mm and 12,000 mm, the trapped person is localized on the first floor. To facilitate the path planning, the user’s positioning coordinates are updated to (−7657.349, 15,607.830, 8200).

(8) $$\mathrm{Homographic}\;\mathrm{Matrix}=\left[\begin{array}{ccc} 1.491e+00 & -3.871e-03 & -3.614e+01\\ 4.791e-01 & 1.079e+00 & -3.505e+01\\ 3.468e-04 & -1.926e-05 & 1.000e+00 \end{array}\right]$$

**Figure 16:**
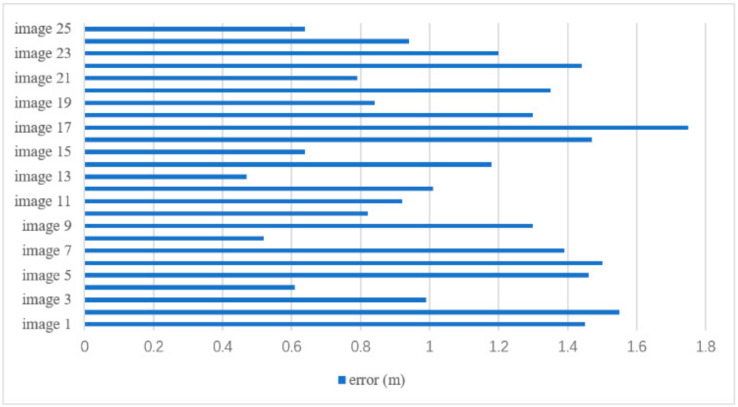
Positioning errors of 25 test images.

For testing of the localization algorithm, 25 images are taken again, and the recognition rate of the model on the test set is 100%. After 25 test images correctly identified, we utilize the algorithm for localization according to the scene of the reference image label and calculate the positioning error, which is shown in Figure 16. According to statistics, the average errors of *x*, *y* and *z* axis are, respectively, 0.555 m, 0.450 m and 0.696 m. The overall estimated error is in the range [0.47, 1.75], of which 44% of the point errors are below 1 m and 92% of the point errors are below 1.5 m. Experiments show that the indoor positioning algorithm applied in this paper can achieve relatively high accuracy.

### 4.3. Indoor Rescue Route Planning

**Figure 17:**
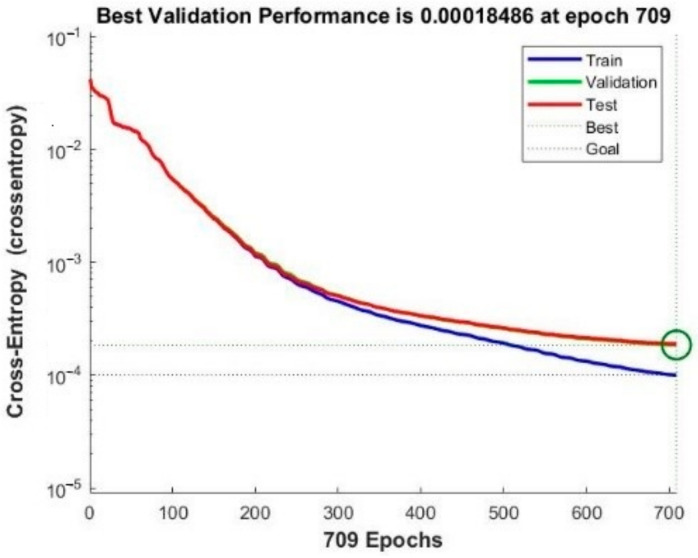
Model performance curve.

**Figure 18:**
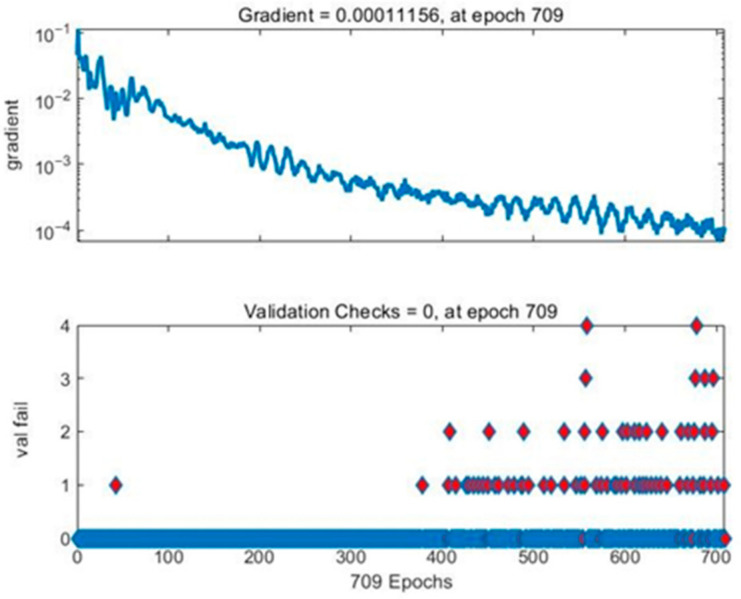
Gradient curve and verification check.

In the path planning module, BP neural network is designed and trained for accelerating the process of path planning. Once the neural network is trained, the resulting model can always be applied for rescue routing of the same building, which indicates that the training cost is relatively low. To ensure the accuracy of the trained path-planning model for calculating optimal rescue route, path samples utilized for establishing planning model of rescue route should be generated as many as possible. Through 100,000 cycle iterations in the path generation module, the generated path samples were scored and a total of 269,637 path samples’ data were finally generated. Making full use of the training data and avoiding overfitting, the cross-validation method is applied for training and validation checks are set as 10. To guarantee the high accuracy of calculating optimal rescue route, the proportion of training set, verification set and test set is, respectively, set as 75%:15%:15% according to [61,62], after which a new test sample is generated additionally for further verification of the trained model. The training effect of neural network is shown in Figure 17 and the gradient curve and verification check are shown in Figure 18.

**Figure 19:**
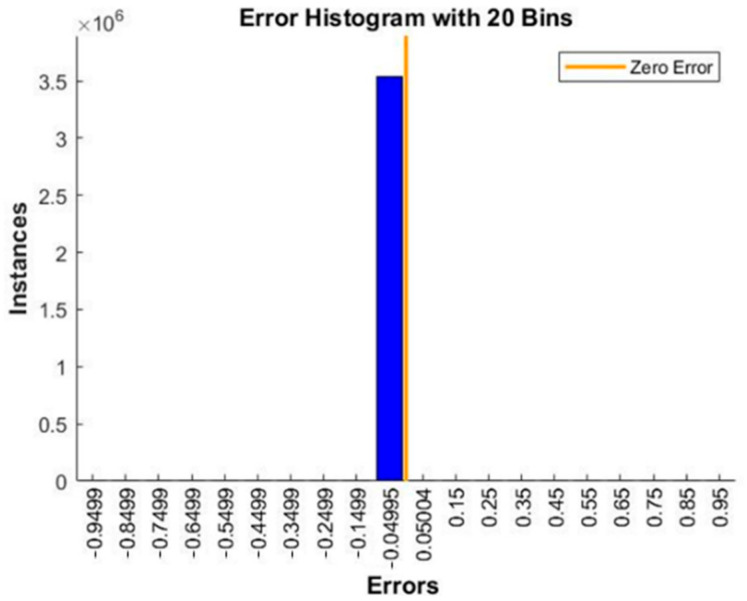
Error histogram.

**Figure 20:**
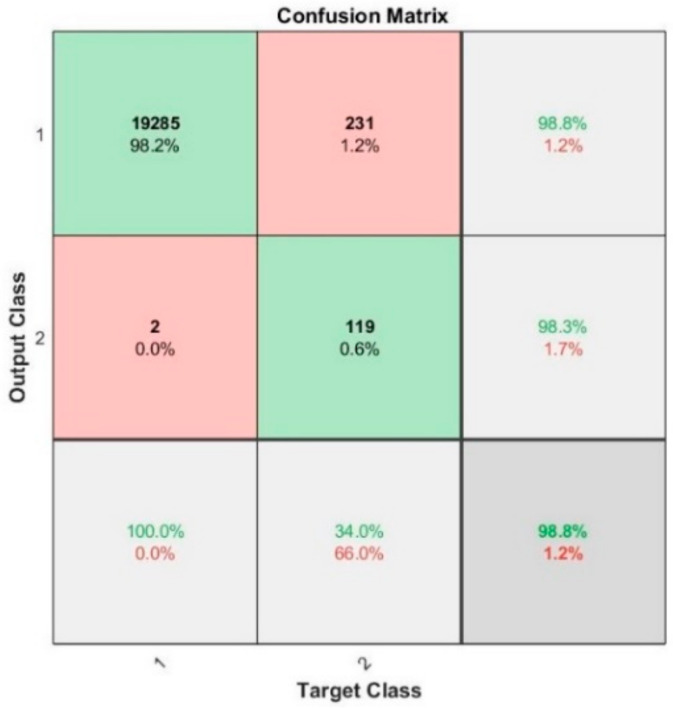
Confusion matrix.

As shown in Figure 19, the training program stops at 709th iteration when the training error reaches the preset threshold. Moreover, for testing the model on the test set, error histogram and confusion matrix are applied to verify the accuracy of the model on the new test set. The total number of the test samples is 19,637, which is newly generated for further verification of the accuracy of the trained path-planning model for calculating optimal rescue route. Figure 20 indicates that the error of the sample is concentrated in the interval [−0.049, 0], within the acceptable range. As shown in Figure 20, regarding the prediction of path nodes as a multi-classification problem, the total accuracy of the program is 98.8%. The data above shows that the neural network has learned the topological structure of the indoor road network with a considerable test set, and the path optimization error rate in the fire scenario is 1.2%.

**Figure 21:**
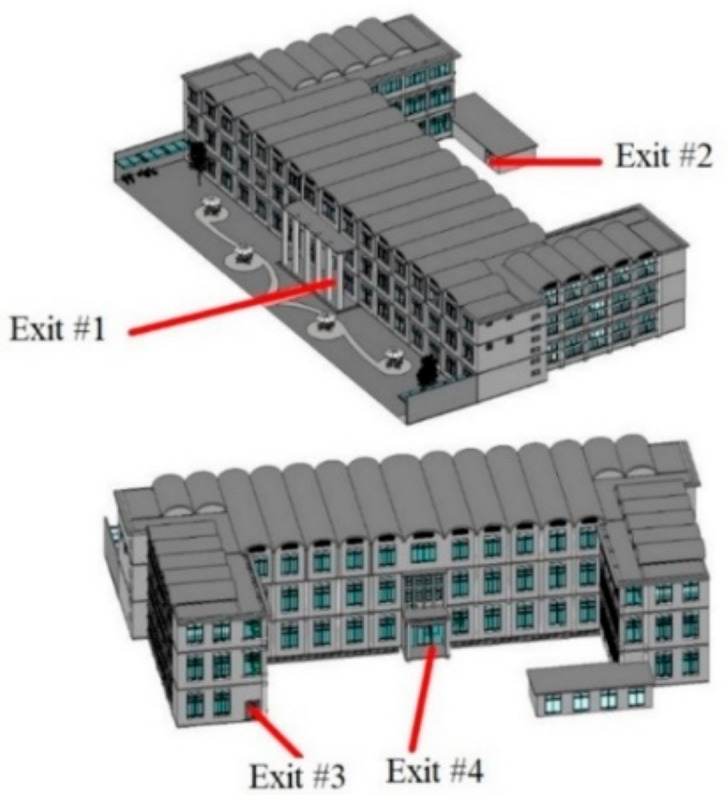
Exits of the building.

**Figure 22:**
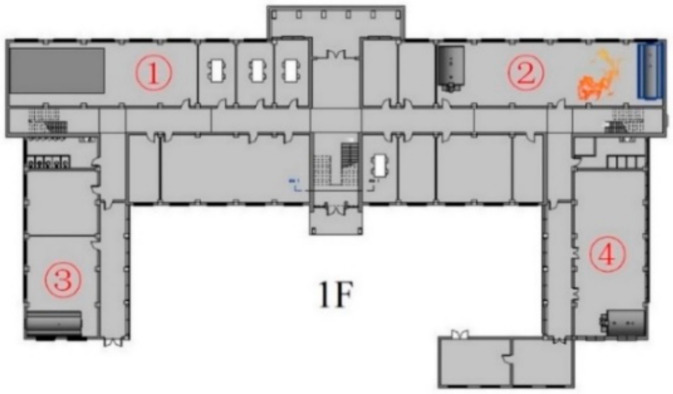
Fire location.

**Figure 23:**
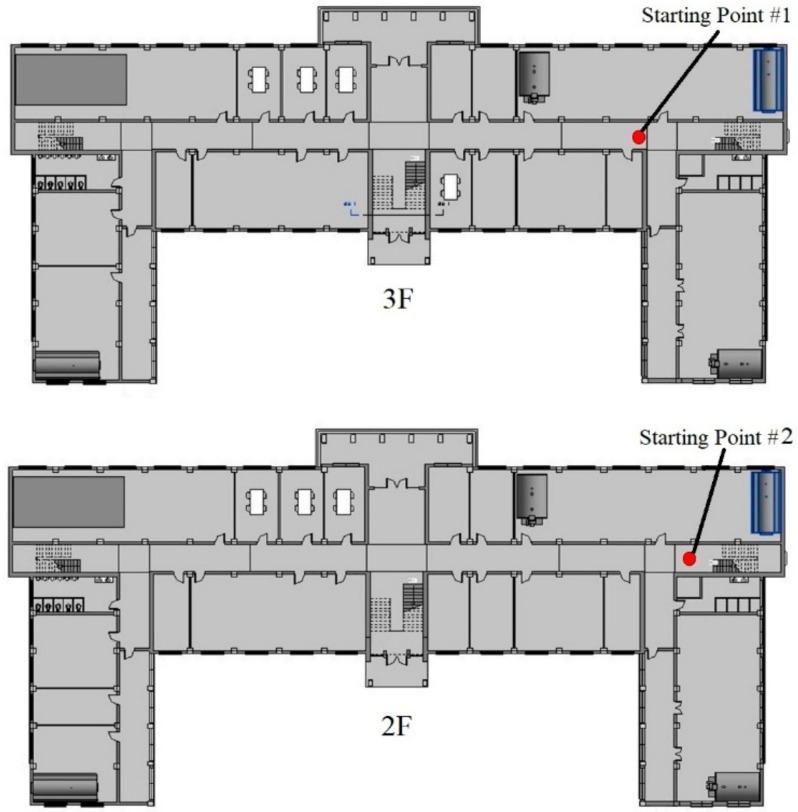
Starting points of evacuation.

After the BP neural network designed for fire emergency path planning has been trained in advance, the fire evacuation framework is verified by case study. In order to better prove the capacity of the path planning module proposed in this paper, the location of the fire is set according to the most unfavorable situation (the fire on the first floor is most unfavorable to all fire evacuees in the building). As for the location of the evacuation personnel, considering the rapid longitudinal spread of smoke along the stairs, an unfavorable starting point of evacuation is set on the right stairway of each floor. The exits of the building, the fire location and the starting place of evacuation are, respectively, shown in Figure 21, Figure 22 and Figure 23.

**Figure 24:**
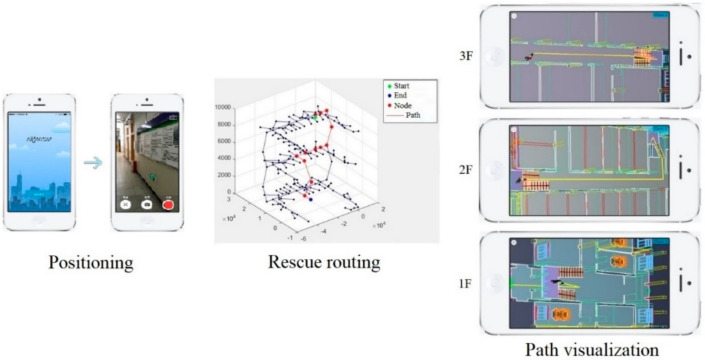
Rescue routing by images.

**Figure 25:**
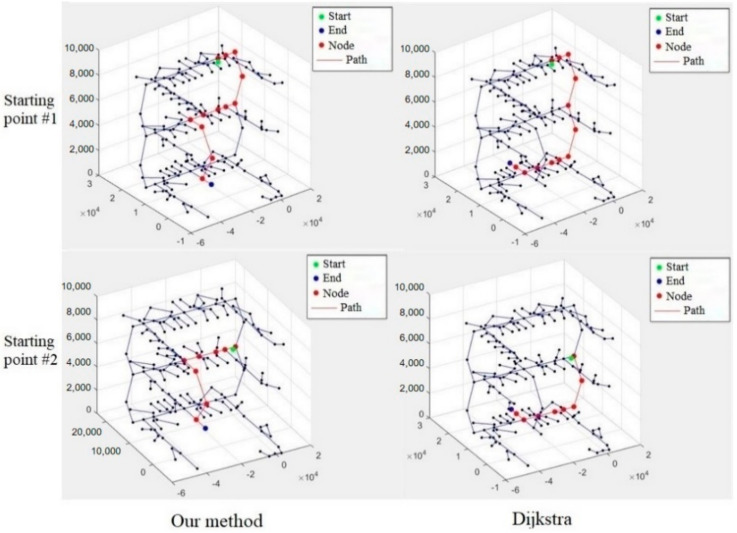
Comparison between our method and Dijkstra.

**Figure 26:**
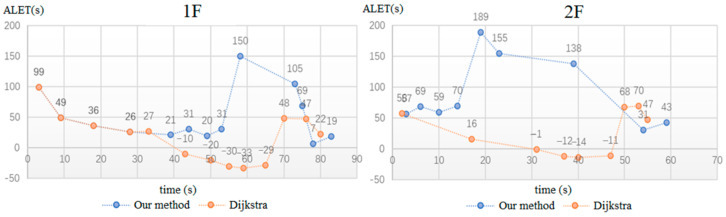
ALET comparison of two algorithms.

Through the real-time photos taken by the users, the starting point evacuation is determined by the proposed indoor positioning method and input into the path planning algorithm, which will be combined with the results of BP neural network for calculating the rescue route (shown in Figure 24). In order to illustrate the safety performance of the rescue route planning, our algorithm is compared with the Dijkstra algorithm, which does not consider the development of fire situation and safety performance of rescue route. In this case, different paths for starting points 1 and 2 are output for comparing, as shown in Figure 25. Comparing the final results of two different algorithms, it is obvious that the rescue route generated by our algorithm took a longer path, increasing the path length by 3.64% and 5.28%, respectively, but avoiding the fire point as much as possible for safer evacuation. For comparison of the above two algorithms, the ALET of them are calculated. As can be seen in Figure 26, the ALET of all nodes in our algorithm is greater than 0, illustrating the better safety performance of rescue route generated.

## 5. Discussion

As a foundation for indoor evacuation navigation in a fire scenario, computer vision technology is adopted for indoor positioning in this research. According to the result shown in Section 4.2, the overall estimated error of indoor positioning is in the range 0.47–1.75, of which 44% of the point errors are below 1 m and 92% of the point errors are below 1.5 m, indicating that the indoor positioning method applied in this paper can achieve relatively high accuracy and provide evacuation navigation with reliable local positioning information.

In order to validate the safety performance of the rescue route planning algorithm, the framework is compared with Dijkstra algorithm, with different paths for different starting points output for comparing. Avoiding the fire point as much as possible, the rescue route generated by our algorithm took longer paths, increasing the path length by 3.64% and 5.28%, respectively. However, the ALET of above two algorithms is calculated and the ALET of all nodes in our algorithm is greater than 0, obviously revealing a better performance of local safety than Dijkstra.

Combining with the proposed indoor positioning method based on computer vision and rescue route planning method considering safety performance of rescue route based on BP neural network, the effect and feasibility of proposed framework is shown in Section 4. It can be seen that the proposed framework successfully utilizes BIM information to extract road network through IFC and obtains the indoor positioning of evacuees, finally providing safe and reliable rescue route for them. It is worth noting that the framework provides an idea of making full use of the rich information in BIM for indoor evacuation in fire scenario. With a positioning error within 1.8 m, as shown in Figure 16, it reveals that the proposed indoor positioning method based on BIM and computer vision technology can reliably ensure the accurate generation of rescue route. On the one hand, if the error within 1.8 m occurs on the same floor, the algorithm will redirect the evacuees to an acceptable room node or corridor node when generating GNM. On the other hand, if the error within 1.8 m occurs across floors, the positioning can be adjusted constantly according to the following scene. Because only the similar scene on different floors can lead to misidentification across floors, it can be corrected by different scenes on different floors.

Compared to the traditional research studies, the proposed framework presents a GNM generation method which properly considers the topological and semantic information of BIM, a passive indoor positioning method which requires no pre-installed equipment and is cheap and convenient to adopt, and a path planning method which is fast and considers the safety performance of rescue route.

## 6. Conclusions

This study proposed a framework for assistance of indoor evacuation in fire scenario, mainly including indoor positioning and rescue route planning by integrating BIM and computer vision. It only needs real-time images to implement the automatic generation of the rescue route, which is cheap and convenient. IFC standard is utilized to automatically generate a GNM of the building, preparing for rescue route planning. To reduce application cost, computer vision technology based on deep learning and transfer learning is applied to localize trapped evacuees without affecting the accuracy of local positioning. By taking ALET into account and utilizing BP neural network, a rescue route planning algorithm with full consideration of local safety performance is proposed, providing a safe and relatively short rescue route for evacuees.

For validation of the framework presented, a hypothetical case study is implemented, indicating that the proposed method can effectively provide a specific route for emergency response. The IFC class/relations extraction is implemented by IFC Java Toolbox toolkit, for generating the topological primitives. In order to obtain the positioning of evacuees, the transfer AlexNet is applied for image recognition based on which the SIFT algorithm and RPnP are utilized, achieving a result that 92% of error points are below 1.5 m. As for the rescue route planning algorithm, the parameters of BP neural network are set by testing and comparing to learn the topological structure of the indoor road network, coming out with the path optimization error rate in the fire scenario of 1.2%. Finally, a case study is illustrated, presenting the local safety performance of the rescue route generated by the proposed framework.

As can be seen, the GNM generation method proposed highlights the rich topological and semantic information of BIM. The positioning method utilizing transfer AlexNet combined with SIFT and RPnP reaches a relatively high accuracy, preparing for rescue route planning. The proposed BP neural network achieves an effect of generating a relatively safe rescue route. In general, the proposed framework can automatically locate the evacuees and provide them with safe rescue route using only images as input. The framework proposed mainly covers the above three aspects of technological innovation for research studies on indoor rescue routing. It provides a simple and convenient way to implement indoor evacuation navigation. When fire hazard occurs, evacuees can obtain a safe and reliable rescue route by taking pictures with their mobile phone.

Future work in this research requires a modification to the cost function in order to include other influential factors in path-planning besides safety state of rescue route. Specifically, different parameters can be set in Formula (4) to compare their influence on evaluating the safety performance of rescue route. The comparison under various conditions (with different parameters) can be made to determine the optimal parameter collocation. Furthermore, further adjustment should be made in consideration of smoke diffusion, directional bias of the movements and congestion by numerous sensors for a safer rescue route planning. Furthermore, the indoor positioning method needs to be improved to prevent the smoke from affecting the positioning effect.

## Figures and Tables

**Table 1:** Comparison between the existing methods and the proposed method.

	The Existing Methods	The Proposed Method
GNM extraction	Less consideration of semantic information;	Full consideration of semantic information;
Indoor positioning	Active; Expensive; Requiring pre-installed equipment;	Passive; Cheap; Without pre-installed equipment;
Rescue routing	Time-consuming;	Fast; Considering safety;

**Table 2:** Indoor elements for GNM.

GNM Elements	Spatial Meanings	Spatial Elements	IFC Entity
Node	Space	Room	IfcSpace
	Horizontal entrance	Door	IfcDoor
Edge	Horizontal path	Corridor	IfcSpace
	Vertical path	Room to door	IfcRelSpaceBoundary
		Corridor to door	IfcRelSpaceBoundary
		Stair	IfcStair

**Table 3:** Elements of GNM.

Gnm Elements	Meaning	Element Name	Ifc Classes	Numbers
Node	Space	Room	IfcSpace	64
	Space	Corridor	IfcSpace	38
	Horizontal protal	Door	IfcDoor	74
	Vertical protal	Stair area	IfcStairFlight	5
Edge	Horizontal route	Corridor	IfcSpace	42
	Horizontal route	Room-to-door	IfcRelSpaceBoundary	74
	Horizontal route	Door-to-corridor	IfcRelSpaceBoundary	74
	Vertical route	Stair	IfcStairFlight	15

**Table 4:** Pixel coordinates after transformation.

Pixel Coordinate	X	Y
Control point#1	2206.489	2988.596
Control point#2	2626.063	3382.856
Control point#3	2668.052	1664.669
Control point#4	2211.204	1673.515

## Data Availability

Not applicable.
